# Multimodal MRI suggests that male homosexuality may be linked to cerebral midline structures

**DOI:** 10.1371/journal.pone.0203189

**Published:** 2018-10-02

**Authors:** Amirhossein Manzouri, Ivanka Savic

**Affiliations:** 1 Department of Women’s and Children’s Health, and Neurology Clinic, Karolinska Institute and Hospital, Stockholm, Sweden; 2 Department of Psychology, Stockholm University, Stockholm, Sweden; City of Hope, UNITED STATES

## Abstract

The neurobiology of sexual preference is often discussed in terms of cerebral sex dimorphism. Yet, our knowledge about possible cerebral differences between homosexual men (HoM), heterosexual men (HeM) and heterosexual women (HeW) are extremely limited. In the present MRI study, we addressed this issue investigating measures of cerebral anatomy and function, which were previously reported to show sex difference. Specifically, we asked whether there were any signs of sex atypical cerebral dimorphism among HoM, if these were widely distributed (providing substrate for more general ‘female’ behavioral characteristics among HoM), or restricted to networks involved in self-referential sexual arousal. Cortical thickness (Cth), surface area (SA), subcortical structural volumes, and resting state functional connectivity were compared between 30 (HoM), 35 (HeM) and 38 (HeW). HoM displayed a significantly thicker anterior cingulate cortex (ACC), precuneus, and the left occipito-temporal cortex compared to both control groups. These differences seemed coordinated, since HoM also displayed stronger cortico-cortical covariations between these regions. Furthermore, functional connections within the default mode network, which mediates self- referential processing, and includes the ACC and precuneus were significantly weaker in HoM than HeM and HeW, whereas their functional connectivity between the thalamus and hypothalamus (important nodes for sexual behavior) was stronger. In addition to these singular features, HoM displayed ‘female’ characteristics, with a similar Cth in the left superior parietal and cuneus cortices as HeW, but different from HeM. These data suggest both singular and sex atypical features and motivate further investigations of cerebral midline structures in relation to male homosexuality.

## Introduction

One of the more controversial questions in the neurobiology of human behavior relates to the mechanisms of sexual orientation. Sexual orientation refers to sexual attraction toward persons of the opposite sex or the same sex. Just like other behaviors, sexual orientation can be viewed as interplay between specific cerebral processes. These processes encompass at least three levels: (1) perception (feeling of attraction triggered by sensory perception), (2) self-identity (feeling that attraction is related to ‘self’), and (3) conscious action towards the desired sex. Most of the hitherto performed studies related to sexual orientation focused on the first level, using various brain imaging methods, and investigating cerebral activation during sexual arousal, elicited by passive viewing of film clips. A majority of these brain imaging studies of cue-induced sexual arousal seem to agree that the cerebral response is invariant to the preferred sex [1–3] and is primarily related to whether the stimulus is from the desired or non-desired sex, although there are also some exceptions [3–6]. In a series of studies of subjects smelling putative pheromones, we also noticed that activation of the anterior hypothalamus in HoM was reciprocal to that of HeM and similar to the activation pattern of HeW [7,8]. Furthermore, recently, Zhou et al. found that smelling a putative male pheromone enhanced the visual perception of male figures in HoM and HeW, but not in HeM, indicating that hypothalamic activation by the male putative pheromone in HoM and HeW had downstream effects on visual perception with a potential impact on selection of sexual partner [9–11].

While intriguing, these studies only imaged perceptional processes, and could, merely reflect learned behavior. However, by indicating a link to structures located along the antero-posterior axis of the brain, several of which have been reported as sexually dimorphic (the thalamus, hypothalamus, amygdala), these studies raised important new questions. One is whether MRI methodology could be used to investigate whether there are any structural brain differences between homo and heterosexual persons. Another important issue, which has not been emphasized in earlier studies, is whether such differences, if existing, reflect a generally ‘sex-atypical’ sexual dimorphism, or, if they are restricted to areas processing sexual cues and arousal, and, thus, confined to the networks mediating sexual behavior. These regions vary in different studies, but do, according to a recent meta analysis primarily include the thalamus, hypothalamus, the pregenual anterior cingulate cortex (pACC), and the perirhinal cortex [12].

Several early post mortem studies suggest structural/histological differences between homosexual men (HoM) and heterosexual men (HeM) along the cerebral midline. Zhou et al., reported that the size of vasopressin neurons was smaller in the suprachiasmatic nucleus of the hypothalamus in HoM compared to HeM LeVay found that the size of the third interstitial nucleus of the anterior hypothalamus was smaller in HoM than HeM, a finding that was later criticized [13], and Allen and Gorski observed a larger cross-sectional area of the anterior commissure [14] among HoM.

Subsequent in vivo brain imaging studies found that the isthmus of the corpus callosum was larger in HoM than HeM [15], and that HoM and heterosexual women (HoW) had a sex-reversed pattern of hemispheric volume asymmetry [8]. More recently, Hu et al. reported increased homogeneity in resting state brain activity (suggested to reflect local functional connections) in mid frontal lobe and decreased homogeneity in the middle and inferior occipital lobe [1].

While congruently suggesting a less pronounced or atypical sexual differentiation of cerebral midline structures, most of these studies investigated a single structure, or used a single metric, and none really addressed the question as to whether the detected differences between homo- and hetero-sexual persons reflected a widespread atypical cerebral sex dimorphism, including several different facets of brain structure, among HeM or restrictive changes around the cerebral midline. Furthermore, to the best of our knowledge, none of these studies combined structural and functional measures to investigate possible coordinated changes involved in encoding sexual preference which would suggest that related neuroanatomical variations are not merely epiphenomena. Several previous reports also relayed on mere comparisons between homo-and heterosexual men, limiting the discussions about sex dimorphism.

In the present study, which is part of a larger effort to elucidate the possible neurobiology of sexual orientation and gender identity, structural and resting state functional MRI have been utilized in tandem to investigate possible cerebral correlates to male homosexuality. We specifically asked ourselves whether the brains of homo and heterosexual men were anatomically different, and how these differences were related to the differences in corresponding measures between HeM and HeW. At variance from our previous investigations [16] data analysis included both cortical thickness (Cth) and surface area (SA), as each of these metrices have been reported to differ between males and females [17–23]. Cth and SA also seem to have different genetic coding [24,25], and, could, thus, be modified independently. Cerebral dimorphism with respect to Cth and SA is supposed to be widespread [18]–therefore we used the entire brain as search space. To further investigate how male sexual orientation was related to cerebral sex dimorphism we also measured volumes of subcortical structures described to differ between men and women–the amygdala, hippocampus, caudate, putamen, and thalamus [17,19,26–29]. The accumbens, which is also of interest in the context of sexual behavior, was not selected as this is a small region difficult to accurately outline on MR images. In addition, assuming that the direction of sexual attraction is inherently related to perception of self, and, thus, should engage the self-referential neuronal networks [30,31], we investigated functional connections within the so-called default mode network (DMN). This network is active during rest and mind wandering, and consists of the pregenual cingulate cortex (pACC), the posterior cingulate and precuneus cortex, the right and left inferior, lateral parietal cortex [32–35]. The DMN was of special interest to investigate also because it covers the majority of structures/regions inferred in sexual arousal and behavior (except for the amygdala, hypothalamus and thalamus), allowing us to explore whether possible changes in structures mediating sexual behavior were corresponded by changes in functional connections between these structures. We also evaluated possible group differences in resting state functional connections from the hypothalamus, and thalamus. This was done through seed region analyses. These regions were of particular interest as they are reportedly sexually dimorphic, and also involved in sexual arousal.

This study design allowed us to examine:

If HoM differed from HeM analogously to HeW, thus suggesting a ‘female typical’ dimorphism, or, to the contrary?

If such differences were located exclusively within the midbrain networks (preferably those mediating sexual behavior and self-referential processing), but not otherwise.

If HoM differed from both heterosexual control groups.

## Material and methods

### Subjects

Thirty homosexual men (age 31±5.5, education 15.8±2.6 years) were investigated together with 35 heterosexual men (age 32.3±6.4, education 16.5±2.6 years) and 38 heterosexual women (age 31.3±6.7, education 17.2±3.0 years). Subjects were recruited through friends and their close circles and local campus advertisements. The authors assert that all procedures contributing to this work comply with the ethical standards of the relevant national and institutional committees on human experimentation and with the Helsinki Declaration of 1975, as revised in 2008. All participants were right handed [36], healthy, and HIV negative. HeM and HeW scored 0–1 and HoM scored 5–6 on the Kinsey heterosexual/homosexual scale (0 = maximally heterosexual, 6 = maximally homosexual) [10,37]. In addition to scoring themselves on the Kinsey scale, the subjects also participated in interviews addressing three dimensions of their sexual orientation (fantasy, romantic attraction, and sexual behavior), divided into consecutive 5-year historical time periods, from age 16 to the present [10]. All decisions about the sexual orientation of subjects were made without knowledge of the MR data. All the participants had stable sexual orientation, which they were aware of before puberty.

Subjects were excluded if they had a previous history of psychosis, personality disorder, sexual dysfunction, gender dysphoria, hypogonadism, HIV infection, paraphilia, or sexual offences, major or bipolar depression, alcohol or substance abuse, chronic psychosocial stress, major life traumas, chronic fatigue, chronic pain, or systemic disease. These exclusion factors were tested for each subject using standard questionnaires described in one of our previous publications [38]. Further exclusion criteria were head trauma and neurological disease. The medical history was taken by a practicing physician (IS). No daily medication was allowed, and none of the participants had previous or ongoing sex hormone medication, except for oral contraceptives in HeW. Possible group differences in psychosocial stress were tested with MBI-GS scores; In addition we compared the groups with regard to anxiety and depression scores using Mongomery-Asberg questionnaires as described previously [39].

The study was approved by the Ethics Committee at Karolinska Institutet, and written informed consent was received from each participant.

### MRI acquisition

Magnetic resonance imaging data was acquired on a 3-Tesla MRI medical scanner (Discovery 3T GE-MR750, General Electric, Milwaukee, Wisconsin) equipped with a 32-channel and 8-channel phased array receiving coil. The 32-channel coil was used for all the sequences; The 8-channel coil was used, in addition, for acquisition of the 3D T1 images (see further) as we found signal inhomogeniety at the frontal and occipital poles when employing the 32-channel coil, not present when using the 8-channel col. The 32-channel coil was preferable for acquisition of the fMRI data. ALL the subjects were investigated with identical protocols. 3D T1-weighted spoiled gradient (SPGR) images were acquired with 1 mm3 isotropic voxel size (TE = 3.1ms, TR = 7.9ms, TI = 450ms, FoV = 24cm, 176 axial slices, flip angle of 12 deg.). MR sequences included resting state functional MRI, (closed eyes, 10 minutes) performed with a gradient echo pulse sequence using a voxel size of 3x3 mm, (TE = 30ms, TR = 2500ms, FoV = 28.8cm, 44 interleaved axial slices, 3mm thickness, flip angle of 90 deg.). Finally, there was a clinical sagital FLAIR: TE/TR = 117/8000, TI = 2255, ETL = 140, ARC acceler. R = 2 x 2 (slice, phase), FoV: 27cm, 224x224, slice thickness, 1.2 mm.

The female controls were tested day 10–14 of the menstrual cycle (all had regular 4 weeks cycles).

### MRI analyses

#### Cortical thickness, surface area, and subcortical structural volumes

The MR volumes were processed using FreeSurfer software version 5.3 as described in our previous studies [19,40]. Both SA and Cth were calculated. Possible group differences were evaluated for each vertex using a 10 mm filter, and age as the nuisance variable (Monte Carlo correction, 5000 permutations, p<0.05) in qdec statistics, Qdec = query design estimate contrast is a software package used to conduct a group analysis of morphometric data with Graphical User Interface (GUI), and is powered by a general linear model-fitter. Because there are age-related changes in Cth, and in some regions there seems to be an age by sex interaction on Cth (the age-cth correlation slope may, thus, be different in males and females), [41], we used the QDEC option of different slope and different intercept. This option takes into consideration the age by gender interaction on Cth and calculates the intercept (= group difference in Cth) at the mean age for both groups.

For further information about the MRI protocol and data analysis, please see [42,43], our previous publications [19,40], and the S1 Supplemental Information.

FreeSurfer pipeline was also used for subcortical segmentations. The subcortical structures of interest were the amygdala, hippocampus, caudate, putamen, and thalamus, because they have in previous studies shown sex differences (see introduction). In addition, we assessed total intracranial volume (ICV). When required, the segmented brain structural masks were modified manually by a rater who was not informed of the identities of the subjects. Data from 10 controls and 5 HoM needed manual corrections. These regarded over estimation of the hippocampus volume at the expense of amygdala (the linea alba border) and the lateral outline of putamen–separation from the claustrum. The same person analyzed all the subcortical volumes.

Ratios between the respective VOIs and the total intracranial volume (TIV), retrieved from the FreeSurfer program, were entered into the statistical analyses. After ensuring that the data were normally distributed, group comparisons of relative structural volumes (VOI/TIV) were performed with one- way ANOVAs using the individual relative values for each type of structure as input values (p < .005, with Bonferroni correction for the ten repeated tests, one for each region). Possible differences between specific groups were tested with Scheffe’s post hoc test (p < .05). The analyses were carried out with PASW Statistics 21 (SPSS Inc., Chicago, IL).

#### Conjunction analysis, cortical thickness and surface area

To investigate whether there were common differences between two groups in relation to the third, conjunctional analyses were carried out as described previously [19,40]; see also [44]. Conjunctional analysis was carried out if the initial analysis o Cth and SA showed differences between HoM and controls, as we were specifically interested in investigating whether HoM had Cth and SA patterns that were more similar to that of HeM than HeW (p<0.05 corrected, after Monte Carlo simulation).

#### Resting state fMRI

Spatial preprocessing of the functional images was performed using SPM8 (Welcome Department of Cognitive Neurology) according to the standardized procedure and by incorporating fieldmap correction. The functional images were slice-time corrected, realigned, and registered to structural T1 SPGR images (acquired with the 32 channel coil) for each participant. After segmenting the individual T1 SPGR images into gray matter, white matter, and cerebrospinal fluid, the gray matter images were used to determine the normalization parameters for the standard MNI gray matter template. At this point, the spatial parameters were applied to the slice-timed and realigned functional volumes that were resampled to 2.0 x 2.0 x 2.0 mm voxels and smoothed with a 6-mm FWHM kernel. Each voxel’s time series was corrected for noise using the SPM standard 128-s high-pass filter combined with AR auto correlation correction modeling. In addition, we employed voxel wise multidimensional regression analysis in a standardized manner to remove artifacts resulting from motion and changes in ventricle and white matter signals [45], by adding 18 movement regressors; 6 parameters obtained from rigid-body head motion correction (SPM 8 statistical package), and their squares and cubes.

Movement correction was conducted also through the use of ICA-AROMA, which automatically identifies and subsequently removes data-driven derived components that represent motion-related artifacts [46]. Head movement > 1 mm was reason for exclusion of further analyses (not needed in these study groups).

ROI analysis was carried out using the SPM 8 package as described in our previous studies [18,47]. The seed regions of interest were placed in the thalamus, and the anterior hypothalamus. The rationale for choice of these specific seed regions was that they have been linked to sexual orientation in previous experimental studies (see introduction). Due to signal loss in the fMRI scans in the mesial amygdala we did not add amygdala seed, although this structure was of interest. The right and left thalamus seed was circular with a 5 mm radius, centered at 10–22–2, and -10–22–2. It covered primarily the pulvinar, because it is an ‘associative’ thalamic nucleus, meaning that most of its input and output relationships are formed with the cerebral cortex, and the centromedial thalamic nucleus because of the previously described differences between HoM and HeM during sexually arousing visual activation [5]. The hypothalamus ROI was a circular 6 mm region, centered at (5 0–7), covering the anterior portion including the preoptic nucleus, region showing a different pattern of putative pheromone activation in our previous studies [7]. The ROI placement was guided by WFU-pick atlas (http://fmri.wfubmc.edu/software/PickAtlas).

For all participants, the average fMRI time course within each seed region was used as the regressor of interest. Individual time series in each seed region was extracted using MarsBar toolbox (http://marsbar.sourceforge.net). The seed region time course for each participant was then regressed voxel-wise against the participant’s fMRI time course using the entire brain as the search space. This approach reveals the strength of functional connectivity with respect to the seed region. Group comparisons were carried out using SPM8, and results are reported at a cluster-level threshold of pFWE < .05.

#### Independent component analysis

After an initial preprocessing with SPM8 (Welcome Department of Cognitive Neurology), the data were analyzed in FSL v5.0 (FMRIB Software Library, Oxford, http://fsl.fmrib.ox.ac.uk/), using a high-pass filter at 100s before running individual independent component analyses (ICA) [48] as implemented in MELODIC (Multivariate Exploratory Linear Decomposition into Independent Components, Version 3.14), with automatic determination of dimensionality. The resulting component maps were then manually classified into components of interest and nuisance components in accordance with the criteria proposed [49–51]. The nuisance components were subsequently regressed out of the original data set using fsl_regfilt.

Group concat-ICA was performed on the entire cleaned dataset, resulting in 22 components. These components were used to run dual-regression analysis and the resulting GLM parameter estimate images were fed into the 'randomise' nonparametric permutation inference in order to test hypotheses about differences between groups in regard to connectivity within networks. In this particular study, we were only interested in the DMN (see introduction for the rationale), which represented one of the 22 independent components. The remaining components represented other cerebral networks [51], and were not used for the further analysis in the present report. The statistical comparisons included age and mean DVARS (Root Mean Square intensity difference of volume N to volume N+1) as nuisance covariates, as described in one of our previous studies [52]. The significance threshold was set at *p* < .05, FWE corrected at cluster level, and possible differences were tested between HeM, HeW, and HoM within the DMN.

## Results

### Demographical data

As expected, the groups differed with respect to the Kinsey scale (p < .0001), but not in age or education (Table 1).

**Table 1:** Demographic data.

	Unit	HeM (N = 35)		HeW (N = 38)		HoM (N = 30)		F(df)-value	*P*-value
		Mean	Sd	mean	Sd	Mean	Sd		
Education	year	32.3	6.3	31.3	6.2	31.4	6.1	0.233*(2*,*100*);	.793
Education	year	16.5	2.7	17.2	3.2	15.7	2.7	1.974*(2*,*100*);	.144
Right D2:D4	ratio	0.97	0.02	1.01	0.02	0.98	0.02	3.678 *(2*,*100*);	.038^**a**^^,^^**b**^
Left D2:D4	ratio	0.98	0.03	0.99	0.03	0.98	0.03	1.958*(2*,*100*);	.147
Kinsey scale		0.47	0.83	0.63	0.94	5.70	0.48	418.2 *(2*,*100*);	.000^c^

^**a**^ Difference between HeM and HeW, *p* = .002

^**b**^ Comparison between HeM and HoM, *p* = .070

^**c**^ Comparison between HeM and HoM, and HeW and HoM *p* < .001

F-values from group comparisons (one way ANOVA). Possible differences in digit ratios between the separate groups were calculated with Scheffe’s post hoc test (*p* < .05).

### Cortical thickness

**Fig 1:**
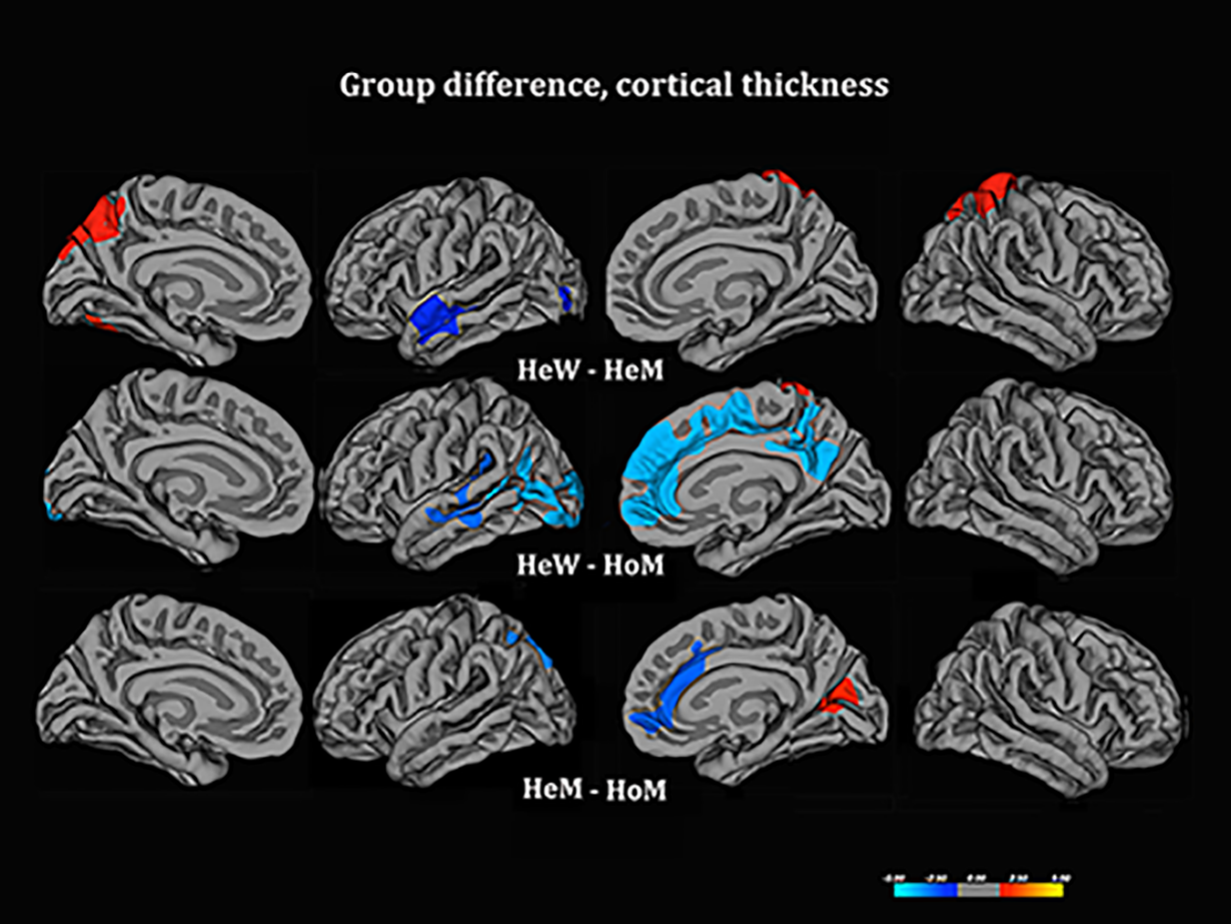
Group comparisons for Cth. The contrasts were calculated at *p* < .05, corrected for multiple comparisons (Monte Carlo permutation), using age as the covariate of no interest. The projection of cerebral hemispheres (MR images of the FreeSurfer atlas) is standardized. Scale is logarithmic and shows–log10(P), with cool colors indicating negative contrast, warm colors positive contrast. The two smaller images at the bottom illustrate Cth in HeM-HoM (left) and HeW–HoM (right) calculated without Monte Carlo correction; they were added to illustrate the HoM differed from both control groups in a similar manner.

**Fig 2:**
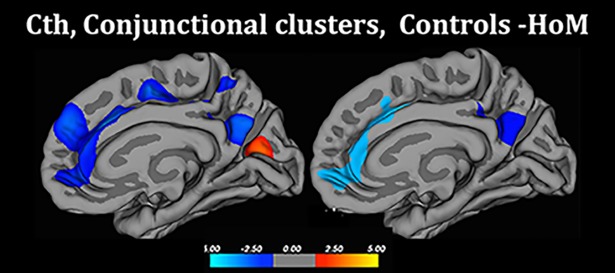
Cortical thickness, conjunctional clusters HoM- Controls. Sagital view of the standard brain MRI (atlas retrieved from the FreeSurfer program’s pipeline), showing regions in which HoM differed from both control groups with respect to Cth. Scale is logarithmic and shows–log10(P), with cool colors indicating negative contrast (thicker cortex in HoM than both control groups), warm colors positive contrast (thicker cortex in control groups).

We first compared the three groups with respect to Cth and SA. Congruent with previous reports, the parietal lobe cortex (including the postcentral gyrus and the right and left superior parietal lobe), and sensory-motor cortex were thicker in HeW than HeM, whereas the left superior/middle temporal gyrus and the left lateral occipital cortex were thinner (Fig 1, Table 2). The left superior/middle temporal gyrus in HeW were thinner also compared to HoM, whereas portions of the sensory motor cortex were thicker in HeW than HoM, with no differences between HoM and HeM. However, there was also a deviation from this ‘sex typical pattern’. First of all, HoM differed from both control groups by having thicker cortex in the right ACC, the superior frontal gyrus and the precuneus (the latter differed only subsignificantly in comparison with HeM), as well as in the left occipito-temporal cortex (covering the extrastriatal body area) (Fig 1, Table 2). Conjunctional analysis [showing shared clusters from the contrast (HoM–HeW) and (HoM–HeM), p < .05 corrected for multiple comparisons after Monte Carlo permutation] confirmed that HoM differed from both control groups by having thicker cortex in the right ACC-midfrontal cortex and right precuneus (Fig 2), but showed no significant cluster in the occipito-temporal cortex. No other conjunctional clusters were detected. A further finding was that HoM differed from HeM, but not HeW, (thus, showing a ‘female’ pattern), in that their left superior parietal cortex was significantly thicker, and their right cuneus was significantly thinner than in HeM, (Fig 1, Table 2).

**Table 2:** Clusters showing significant group difference in cortical thickness.

Cluster	HeW-HeM (positive -log10(p) values) HeM-HeW (negative -log10(p) values)			HeW-HoM (positive -log10 (p) values) HoM-HeW (negative -log10(p) values)			HeM-HoM (positive -log10 (p) values) HoM-HeM (negative -log10(p) values)		
	Maximum vertex-wise -log10(p)	Cluster size, cm^2	Talairach Coordinates	Maximum vertex-wise -log10(p)	Cluster size, cm^2	Talairach Coordinates	Maximum vertex-wise -log10(p)	Cluster size, cm^2	Talairach Coordinates
**R superior + inferior parietal + postcentral gyrus**	4.4	14.1	18–46 57	2.5	11.0	5 2 50			
**L superior + middle temporal gyrus**	-4.2	14.3	-58–25–7^a^	-4.5	34.9	-48–25–7^b^	-3.8	25.3	-55–58 5†
**L precuneus + superior parietal cortex**	2.5	10.0	-8–68 50				-3.4 -2.6	14.7 10.4	-7–59 18 -24–63 28
**L lateral occipital cortex**				-2.6	13.7	-12–67–18	-2.6	11.8	-48–64 1
**R superior and rostral frontal cortex**				-4.8	60.4	14 39 16^c^	-3.8	14.9	15 33 17
**R cuneus, pericalcarine cortex**				*3*.*3*	*22*.*2*	*26–87–8*		12.6	4–71 10

Statistical threshold is *p* < .05, corrected for multiple comparisons (according to Monte Carlo permutations). Demeaned age was used as a nuisance covariate. The filter was 10 mm. The Talairach’s coordinates indicate location of maximum difference, the ‘Region’ column describes the coverage of the respective cluster. Italics indicate clusters calculated at *p* < .05 uncorrected.

R = right; L = left

^**a**^ covers the L temporo-occipital cortex

^**b**^ covers also the lateral occipital lobe

^**c**^ covers also the precuneus

#### Post hoc analyses: Cortico-cortical covariations in Cortical thickness

Several recent studies have found that cerebral functional networks have an intrinsically cohesive modular structure where the modules are composed of functionally as well as anatomically related brain regions, and these networks can be identified by maps of covariance [53]. To investigate whether the observed differences between HoM and the controls were interrelated, post hoc cortico-cortical covariation analyses were performed using the two significant clusters detected in conjunctional analyses (right midfrontal-cingulate cluster and right precuneus cluster, Fig 2) as seed regions (p < .05 after Monte Carlo correction for multiple comparisons), as previously described [40]. In short, the mean Cth from each region of interest (ROI) was first extracted in each subject. Next, the data from each seed ROI was used as covariate of interest to test possible group difference in the covariation pattern between the respective seed region and the rest of the brain. Possible differences in covariation patterns were tested between HoM and HeM, HoM and HeW, and between HeW and HeM (qdec statistics within the FreeSurfer software, p < .05 after Monte Carlo correction for repeated comparisons). HoM showed a significantly stronger correlation compared to both HeM and HeW between the Cth in the precuneus seed and the anterior and mid cingulate, the occipital cortex, the inferior frontal and insular cortex, S1 Fig, S1 Supplemental Information). No difference was detected between HeM and HeW, and no group differences were found with respect to the covariation pattern from the midfrontal-cingulate seed.

#### Post hoc analyses: Correlation between Kinsey scores and cortical thickness

To assess whether the differences in Cth between homo and heterosexual men were specifically linked to sexual orientation, we carried out post hoc analyses including all three study groups (HoM, HeM, and HeW), and testing whether there was any significant correlation between Cth and Kinsey scores. Using the qdec statistics, Kinsey scores were employed as the covariate of interest, (age was a nuisance covariate) in relation to Cth in each vertex of the brain (p < .05 with Monte Carlo correction for multiple comparisons). The analysis showed a significant positive correlation between Kinsey scores and the Cth in the occipito-parietal and precuneus cortex (S2 Fig, S1 Supplemental Information). In addition, there was a sub significant (p < .05 uncorrected) cluster indicating a positive correlation between Kinsey scores and Cth of the ACC. No clusters indicating inverse correlations were detected.

### Surface area

**Fig 3:**
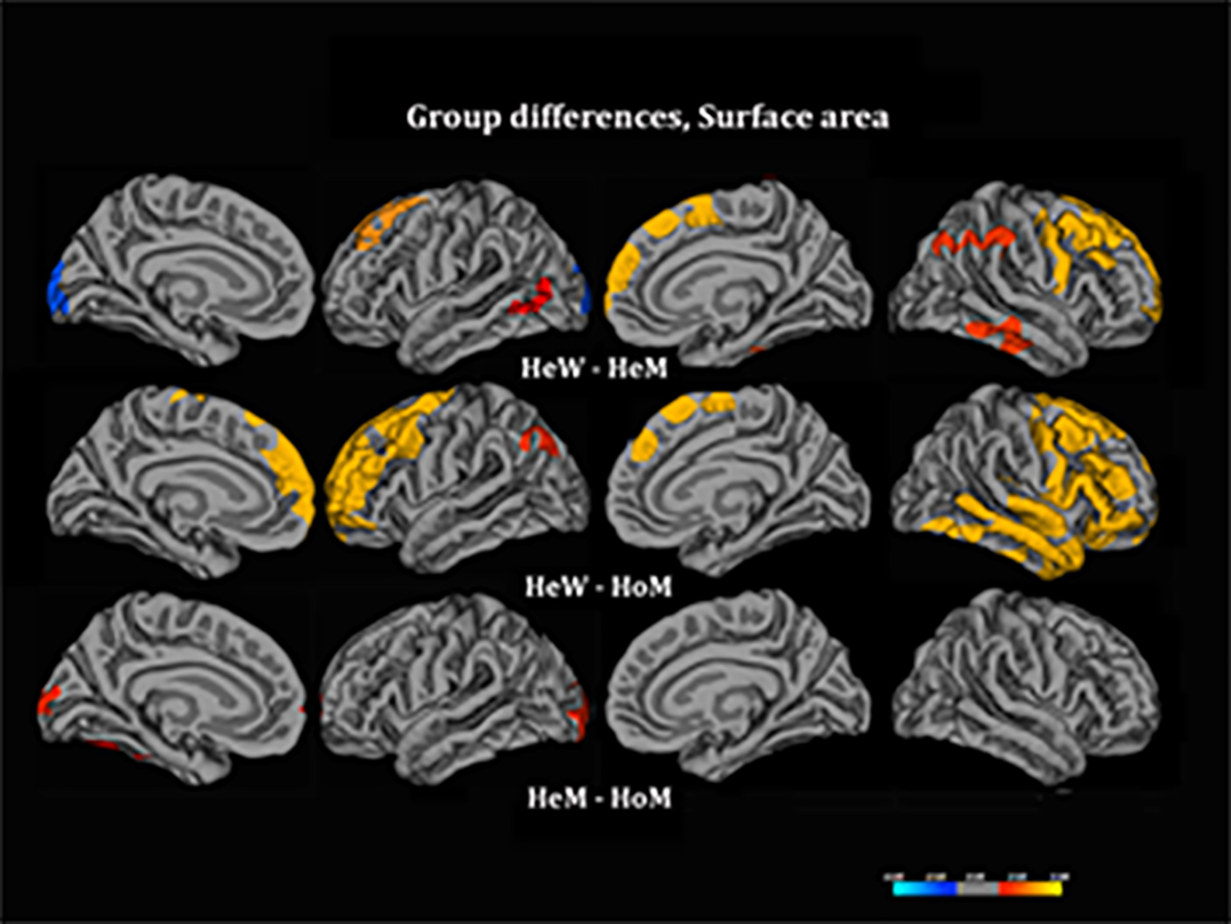
Group comparisons for surface area. The contrasts were calculated at *p* < .05, corrected for multiple comparisons (Monte Carlo permutation), using age as the covariate of no interest. The projection of cerebral hemispheres (MR images of the FreeSurfer atlas) is standardized. Scale is logarithmic and shows–log10(P), with cool colors indicating negative contrast, warm colors positive contrast. The two smaller images at the bottom illustrate Cth in HeM-HoM (left) and HeW–HoM (right) calculated without Monte Carlo correction; they were added to illustrate the HoM differed from both control groups in a similar manner.

Interestingly, and in contrast to the findings on cortical thickness, SA was larger in HeW than in both male groups over larger portions of the frontal, and temporal lobes and, to a lesser extent, the parietal cortex (Table 3 and Fig 3). No significant difference was detected between HoM and HeM with exception for a small cuneus-cluster showing greater SA in HeM compared to HoM and HeW, without significant difference between the two latter groups.

**Table 3:** Clusters showing significant group difference in surface area.

Cluster	HeW-HeM (positive -log10(p) values) HeM-HeW (negative -log10(p) values)			HeW-HoM (positive -log10 (p) values) HoM-HeW (negative -log10(p) values)			HeM-HoM (positive -log10 (p) values) HoM-HeM (negative -log10(p) values)		
	Maximum vertex-wise -log10(p)	Cluster size, cm^2	Talairach Coordinates	Maximum vertex-wise -log10(p)	Cluster size, cm^2	Talairach Coordinates	Maximum vertex-wise -log10(p)	Cluster size, cm^2	Talairach Coordinates
**L superior frontal cortex +L pars opercularis+** **L rostral middle frontal cortex**	3.3	23.8	-10 45 38	7.67.6	40.440.4	-18 60 6–18 60 6			
**L lateral occipital + cuneus**	-2.5	12.4	-13–92 21^a^				2.4	21.7	-5–91–16
**R superior frontal (middle frontal + precentral) cortex**	3.1	12.1	43–2 50	4.2	18.7	38 19 45			
**R superior, middle temporal cortex** **R caudal middle frontal + Orbitofrontal cortex**	3.0	36.0	46 6–27	-6.5 -4.3 -3.5	33.0 18.5 41.9	57–7–7 38 19 45 28 29–11			

Statistical threshold is *p* < .05, corrected for multiple comparisons (according to Monte Carlo permutations). Demeaned age was used as a nuisance covariate. The filter was 10 mm. The Talairach’s coordinates indicate location of maximum difference, the ‘Region’ column describes the coverage of the respective cluster. Italics indicate clusters calculated at *p* < .05 uncorrected.

R = right; L = left

^**a**^ Covers fusiform gyrus, part of the inferior temporal gyrus

In summary, HoM displayed both sexes reversed dimorphism (in the left parietal cortex and right cuneus), and singular features (differing from both male and female controls with respect to ACC/superior frontal and precuneus cortex). Both findings were detected around the cerebral midline and only for Cth. To the contrary there was no difference between HoM and HeM with regard to SA.

### Subcortical volumes

The data from subcortical analyses are presented in Table 4. The total brain volume was significantly smaller in HeW compared to the two male populations, which did not differ from each other. Significant group differences were detected in the caudate, hippocampus and thalamus, but not in the amygdala and putamen, Table 4, one-way ANOVA). The relative thalamus volumes (corrected or the total intracranial volume (thalamus/ICV ratio) in HoM were smaller than in HeW.

**Table 4:** Structural volumes.

Structural Volumes (cm3)	HoM	HeM	HeW	P and *F*(*df)* values
L caudate	4.2±0.5	4.2±0.5	3.9±0.4	p = .031 F = 6.2(*2*,*100)*
R caudate	4.2±0.5	4.2±0.5	4.0±0.4	p = .002 F = 6.8(*2*,*100)*
L putamen	5.3±0.5	5.4±0.7	4.8±0.6	p = .400 F = 0.9(*2*,*100)*
R putamen	5.2±0.5	5.2±0.6	4.7±0.6	p = .401 F = 0.8(*2*,*100)*
L hippocampus	4.2±0.5	4.3±0.4	3.9±0.4	p = .026 F = 3.8(*2*,*100)*
R hippocampus	4.2±0.6	4.4±0.6	4.1±0.3	p = .003 F = 6.0(*2*,*100)*
L amygdala	2.0±0.2	2.0±0.2	1.7±0.2	p = .400 F = 0.7(*2*,*100)*
R amygdala	2.1±0.3	2.0±0.3	1.8±0.2	p = .480 F = 0.8 (*2*,*100)*
L thalamus	7.1±0.4	7.4±0.6	6.8±0.6	p = .002 F = 6.4(*2*,*100)*
R thalamus	7.2 ±0.7	7.5±0.7	6.7±0.6	p = .043 F = 3.2(*2*,*100)*
TIV	1625.5±123.0	1638.4±129.3	1426.5±117.7	p = .0001 F = 37.95(*2*,*100)*

TIV = total intracranial volume; P-values for structural volumes were based on calculations of ratios between the respective structural volume and the TIV.

HeW had smaller TIV than both male groups; There was no difference between HeM and HoM in TIV. Right caudate volume/TIV was larger in HeW compared to HeM, p = .001 and HoM, p = .020; The corresponding p-values for the left caudate/TIV were .003 and .018. The right hippocampus volume/TIV was larger in HeW than HeM (p = .007), and HoM (p = .007). For the left hippocampus/TIV the corresponding p-values were .036 and p = .063. There was no significant difference between HeM and HoM in the caudate/TIV or hippocampus/TIV. The left thalamus volume/TIV was smaller in HoM compared to HeW (p = .003) but not to HeM (p = .053). The corresponding p-values for the right thalamus volume/TIV were .044 and .420. No significant difference was detected between the two control groups in the thalamus volumes.

The caudate volume and the hippocampus volumes were larger in HeW compared to both HeM and HoM, with no difference between the two latter groups. Thus, with respect to structural volumes, there were no signs of a sex atypical or singular pattern in HoM, despite the threshold of *p* < .05 without Bonferroni correction. As shown in Table 4, the main results of the subcortical volume analysis showed a similar pattern when correcting for the 10 repeated analyses (thus using *p* < .005); we regarded, however, such correction unnecessary given the primary hypothesis about sex differences in all the regions selected for analyses, implying that differences between HoM and HeM could be expected in these same regions.

### Resting state functional connectivity

**Fig 4:**
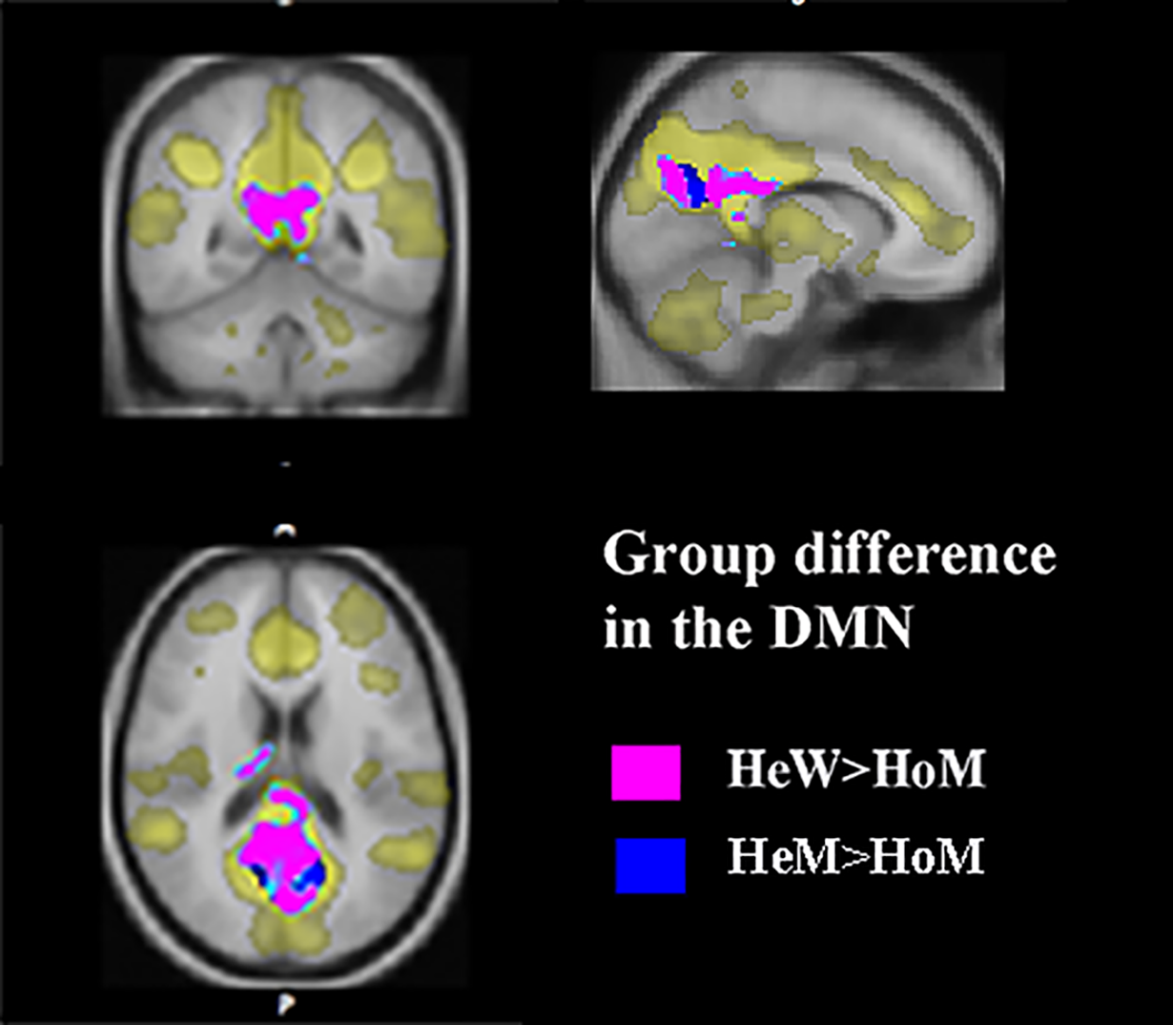
Group differences in the default mode network (DMN). The network (DMN) is depicted in yellow. Decreased connectivity in HoM compared to HeW is shown in pink, decreased connectivity in HoM compared to HeM in blue. Clusters calculated at p<0.05 FEW corrected. There were no significant differences among the controls. Cluster coordinates (MNI) in the figure are X = 10, Y = -42, Z = 28.

HoM differed from HeM as well as HeW with respect to DMN connectivity, with weaker connections in a cluster covering the posterior cingulate, and precuneus, (MNI co-ordinates for the maximum difference, 4–26 35, p < .05 FWE corrected, cluster size 4,2 cc for HeM>HoM comparison and 1.1 cc for the HeW-HoM comparison, Fig 4. HoM also differed from both control groups with respect to thalamus connectivity, and showed stronger connection from the thalamus seed region to the ipsilateral dorsomedial thalamus and contralateral pulvinar and also the anterior hypothalamus, (Table 5, Fig 4). No significant differences in thalamus connectivity were detected between the HeM and HeW, and all three groups displayed a similar connectivity pattern from the hypothalamus seed, with significant clusters in the anterior hypothalamus, both caudate nuclei, and the superior colliculus.

**Table 5:** Group differences in resting state functional connectivity from the selected seed regions.

Seed ROI	Significant connection to:	HeM–HoM			HeW–HoM			HeW–HeM		
		Peak level Z	Size cm^3	Co-ordinate	Peak level Z	Size cm^3	Co-ordinate	Peak level Z	Size cm^3	Co-ordinate
**Left** t**halamus**	Left medio dorsal thalamic nucleus +anterior hypothalamus	-5.2	3.2	-14–20 0 8–10 2	-4.7	1.0	-10–20 2	NS		
**Right thalamus**	Right medio dorsal thalamic nucleus +left pulvinar + anterior hypothalamus	-4.3	6.5	12–20 4 -10–12 2	-6.6 -4.1	2.0 0.5	10–22 4 -20–30 4	NS		
**Hypothalamus**	NS	NS			NS			NS		
		**HeM**			**HeW**			**HoM**		
**The anterior hypothalamus**		inf	27.0	8–4–4	inf	32.0	8–4–4	inf	25.0	8–4–4

Clusters were detected at *p* < .05 FWE corrected at cluster level. Negative Z-values indicate significant differences when running the respective contrast in opposite direction. The thalamus clusters showing significant difference between HoM and HeW and HeM were located in the dorsomedial, dorso lateral thalamus and covered also the pulvinar.

## Discussion

In the present study we investigated whether there are any differences between HoM, HeW, and HeM with respect to various measures of cerebral anatomy and functional connectivity, reported to differ between men and women. A key question was whether such differences, if present, were global, or restricted to cerebral nodes mediating self-referential sexual arousal. The generated data confirmed previous findings of regional sex dimorphism among the heterosexual controls in Cth, SA, and in structural volumes of the hippocampus, and caudate. Whilst HoM showed ‘male’ typical features in SA and structural volumes, the Cth reveled both ‘female’ (thicker left superior parietal cortex and thinner right cuneus cortex compared to HoM) and singular characteristics (thicker ACC, frontopolar and precuneus cortex than in both HeM and HeW). The singular features seemed coordinated, given the significantly stronger cortico-cortical covariations between the precuneus seed ROI and the frontal cortex in HoM compared to both control groups (S1 Fig). These presumably coordinated singular features in HoM were associated with a similarly singular functional hypo-connectivity between the precuneus and the rest of the DMN, combined with an intra-thalamic and thalamo-hypothalamic hyper-connectivity.

Considering that the three groups differed only with respect to their gender and sexual orientation, it is plausible to consider that the potential neurobiological substrates of male homosexuality may be located primarily in regions around the cerebral midline. The data are of interest, because these particular regions (hypothalamus, thalamus) are known to process sexual arousal [12,54,55] and the self-other distinction (the preunneus, and ACC), [30,56]. The present findings argue against a ‘general cerebral feminization’ in HoM. The observed difference in Cth and functional connectivity in the precuneus, which was detected in relation to both female and male controls, deserves special comment, as it arguably might be of relevance for sexual behavior. Precuneus is a multi-associative node with strong reciprocal cortical connections to the cuneus, and to the occipito-temporo parietal junction—*regions processing body cues*. In addition, precuneus is connected to the ACC- a region believed to processes perception of self [31] and to subcortical structures mediating sexual arousal (the pulvinar, dorsomedial thalamus, and hypothalamus), [12,54,55,57]. Precuneus may, thus, represent a pivotal hub for *the processing of choice selective sexual behavior*. Whether the coding of same sex attraction vs. opposite sex *un*- attraction guiding this behavior occurs at the level of precuneus, or at several parts of the aforementioned networks is not evident from the present data. Neither do we know the cause to the detected difference between HoM and controls.

Cth reflects neuronal size, number, dendritic connections in cortical column [58] and can serve as a proxy marker of the integrity of the cerebral cortex. In male controls, the thickness of the parietal and superior frontal lobe cortex is reported to be smaller than in female controls), and inversely correlated to testosterone levels [19,59–61]. In contrast, the left superior temporal and occipital cortices are, according to several studies, thicker than in female controls, and positively correlated to testosterone levels [21]. It is, therefore, possible that the thicker parietal and frontal lobe cortex, as well as the thinner cuneus cortex in HoM could reflect regional hypoandrogenization. Given the normal testosterone levels in our HoM (S1 Supplemental Information, S1 Table), such a tentative hypoandrogenization might mirror a weaker androgen receptor expression or function [62]. Again, the available data do not allow further speculations about possible hormone effects. Cth may undergo dynamic changes, and is reported to increase, as well as decrease with training and learning of a certain task [63]. Learning effects are, however, expected to be associated with an increased, rather than decreased functional connectivity, and are, thus, unlikely to be a major factor behind the present observations. One could speculate that a combined thickening of the parietal cortex, precuneus and ACC could be associated with a variant coding of sexually arousing body cues in relation to self, and perhaps also with a weakened connectivity between the precuneus and the other nodes of the DMN—the ACC and the parietal cortex. Theoretically, it is possible that impaired inhibitory inputs from the precuneus to the pulvinar could have led to a more pronounced connection between the pulvinar, the dorsal medial thalamus, and the anterior hypothalamus, that was observed in our HoM.

### Methodological considerations

The present findings relate only to male homosexuality and cannot be extrapolated to female homosexuality, which requires separate studies. Standard methods were utilized in this study, as described in several of our previous studies [40,47] and the sample size should be considered sufficient to allow conclusions about group differences [64]. In an earlier study from our group, thinning of the cuneus was observed as well as a reduction in thalamus volume in HoM [16]. This study relied on seed region analyses, which provided average thickness values from relatively large cortical regions and, while being largely congruent with the present findings, the data were generated from considerably smaller populations, and are, therefore, not fully comparable. The present study is also regarded as more complete, as it encompassed both structural and functional connectivity analysis. The reason to include functional connectivity analysis was to investigate whether possible structural differences between HoM and controls had any correlates in functional resting state connections in the networks connecting these regions. Such a scenario would strengthen the hypothesis about an involvement of midline networks in the biology of sexual orientation. To address this issue, it is preferable to investigate the entire networks inter connecting the structures showing possible difference between HoM and controls, rather than delineating separate seed regions corresponding to each anatomical structure showing group difference in structural volumes. In this respect it is of interest that the cortico-cortical covariation analysis showed clusters differing HoM and controls in areas well corresponding to those showing a less pronounced functional connectivity.

The DMN cluster included a portion of cerebellar vermis (Fig 4). Part of the vermis, is usually not included in DMN, although is was included in the DMN in some studies [65]. Considering that the clusters showing significant group differences in the present study were strictly incorporated in the classical DMN regions (pCC and precuneus), not in the cerebellum, why choose accept the the DMN component without a further elaboration, (detected at a pFWE corrected < 0.05).

Although the amygdala has been cited as a structure involved in sexual arousal we chose not to use it as a seed region in the Rs-fMRI analysis. The rationale was the increased susceptibility artifact found at 3 Tesla in this specific region, especially its medial portion, which limited the reliability of this measure.

Considering the potential impact of the results, it is essential to discuss alternative explanations. One is the possibility of bias regarding the control populations, since some differences were detected in relation to both HeM and HeW. Arguing against such a bias is that a subsample of this control sample has also been used in relation to other study groups applying an identical methodology [e.g., occupational stress, [40]], producing entirely different results. Another issue worth a comment is that some HeW were using oral contraceptives. However, if anything, this would increase the variance, and lead to a thicker rather than thinner frontal cortex in female controls [66] compared to HoM, which was not the case. In addition, the present differences were detected in relation to both male and female heterosexual controls.

We detected normal testosterone levels, and also estrogen levels among HoM. We did not measure plasma sex hormone levels in controls. However, the majority of controls participated also in another study for which we collected testosterone and estrogen levels using saliva tests (Salimetrics, Penn State, USA). The levels were within the normal range for each control but are not presented as the method was different.

Increased Cth along the ACC and mesial prefrontal cortex has been detected in some populations with functional autism spectrum disorders [67]. None of our homo or heterosexual subjects had such diagnoses, and there were no differences between HoM and HeM with respect to Social Responsiveness Scores, which had been obtained for another study (S1 Supplemental Information). We did not collect data on sexual activity, and it is theoretically possible that the groups differed in this regard. There is, however, to the best of our knowledge, no support in the literature for sexual activity inducing structural changes along the cerebral midline. While this is the first multimodal MRI study to suggest a link between cerebral networks and male homosexuality, the present results do not distinguish environmental, genetic, and hormonal effects, [the HoM group had normal testosterone levels, as well as estrogen levels (S1 Supplemental Information, S1 Table)]. This important issue would be interesting to address in specially designed longitudinal studies.

## Conclusion

The present observations suggest that neuronal networks involved in sexual arousal and self-referential processes (located along the cerebral midline) may be linked to male homosexuality. To what extent the detected differences from controls mirror inherent or acquired processes requires further investigation to elucidate.

## Supporting information

S1 Fig Group differences in cortico-cortical covariations of Cth from the precuneus ROI. The scale is is logarithmic and shows log10(P); Warm colors indicate positive contrast and thus greater covariation in HoM than controls (HeM and HeW). Clusters calculated at p < .05 after Monte Carlo correction are superimposed on a standard MRI brain
(TIF)

S2 Fig Correlation between Kinsey scores and cortical thickness. Sagittal view of the standard brain MRI (atlas retrieved from the FreeSurfer program’s pipeline), showing regions in which Kinsey scores were significantly correlated with Cth. Scale is logarithmic and shows–log10(P), with warm colors positive correlations (thicker cortex, higher Kinsey score), cool colors indicating negative correlation.
(TIF)

S1 Supplemental Information (DOCX)

S1 Table Scores in a test of social functions. (DOCX)

S2 Table Hormone levels in HoM (n = 30). (DOCX)
