# Supplementary figures and images for: Multimodal MRI suggests that male homosexuality may be linked to cerebral midline structures
Source: PLoS One. 2018 Oct 2;13(10):e0203189. doi: 10.1371/journal.pone.0203189 (PMC6168246; doi:10.1371/journal.pone.0203189)

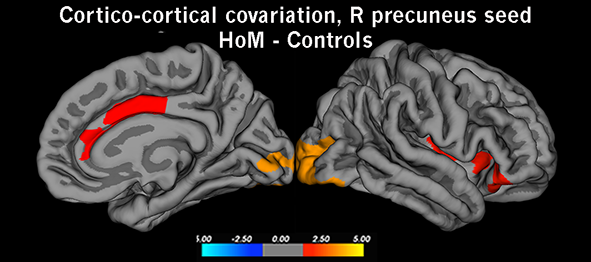

Supplement: S1 Fig — The scale is is logarithmic and shows log10(P); Warm colors indicate positive contrast and thus greater covariation in HoM than controls (HeM and HeW). Clusters calculated at p < .05 after Monte Carlo correction are superimposed on a standard MRI brain (TIF) [file pone.0203189.s001.tif]

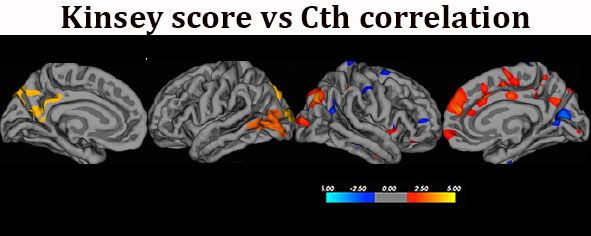

Supplement: S2 Fig — Sagittal view of the standard brain MRI (atlas retrieved from the FreeSurfer program’s pipeline), showing regions in which Kinsey scores were significantly correlated with Cth. Scale is logarithmic and shows–log10(P), with warm colors positive correlations (thicker cortex, higher Kinsey score), cool colors indicating negative correlation. (TIF) [file pone.0203189.s002.tif]
