# Supplementary material for: Multimodal MRI suggests that male homosexuality may be linked to cerebral midline structures
Source: PLoS One. 2018 Oct 2;13(10):e0203189. doi: 10.1371/journal.pone.0203189 (PMC6168246; doi:10.1371/journal.pone.0203189)
Supplement: S1 Supplemental Information — (DOCX) [file pone.0203189.s003.docx]

# Supplemental information

**Cortical thickness andsurface area**

**A** Calculation of surface-based anatomical measures was produced by reconstructing models of white matter (WM) and gray matter (GM) surfaces from MR volumes. The reconstruction of the MRI images was inspected visually after the Talairach transformation, after the skull striping, and after the surfaces had been built and the volumes labeled. Necessary corrections were made after each inspection, including correcting erroneous skull striping by adjusting watershed parameters or manually editing out the skull tissue and adding control points to normalize intensity for erroneous WM surface reconstruction.

Possible group differences in Cth and SA were evaluated for each vertex, using age as the nuisance variable, and after employing Monte Carlo correction (5000 permutations). This method of correction for multiple comparisons creates random noise fields and detects the clusters that appear at certain probability and size thresholds. Following 5000 such iterations, we calculated the frequency for how often a simulated cluster’s value exceeded the value from the true data analysis, which was used to determine the results that were significant at the level corrected for multiple comparisons (p was set to <0.05 corrected). Demeaned age was used as a nuisance variable, and the vertex-by-vertex group comparison (10 mm filter) was carried out in the qdec analyses of Cth and SA.

**Segmentation of the subcortical volumes**

Subcortical segmentations generated with FreeSurfer (Fischl et al. 2002, 2004) were used to calculate the volumes of five subcortical brain structures: amygdala, hippocampus, caudate nucleus, putamen, and thalamus. These were chosen because they typically show sex differences in volume (Savic and Arver 2011, 2014; Lentini et al. 2013). In addition, we assessed total intracranial volume (ICV). Subcortical segmentation generated with FreeSurfer software (www.surfer.nmr.mgh.harvard.edu**)** was used to calculate the volumes of five subcortical brain structures: the amygdala, hippocampus, caudate, thalamus and putamen. When required, the segmented brain structural masks were modified manually by a rater who was not informed of the identities of the subjects. Data from 10 controls and 5 HoM needed manual corrections. These regarded over estimation of the hippocampus volume at the expense of amygdala (the linea alba border) and the lateral outline of putamen – separation from the claustrum. The standard procedure employed has been described in detail previously (Fischl et al., 2002; Fischl et al., 2004) (Savic, 2013)**,** The same person analyzed all the subcortical volumes.

Using SPSS Statistics 21 (SPSS Inc., Chicago, IL), we performed multivariate ANOVA of regional volumes corrected for ICV and age, to compare trans men to cis controls at session 1. As we had a primary hypothesis that in HoM the subcortical volumes of these regions would follow more of ‘a female than male pattern’ no Bonferroni correction ws employed (*p*<.05).

**Resting state fMRI**

Spatial preprocessing of functional images was performed using SPM8 (Welcome Department of Cognitive Neurology, http://www.fil.ion.ucl.ac.uk/spm/). The functional images were slice-time corrected, realigned, and registered to structural T1 SPGR images for each participant. After segmenting the individual T1 SPGR images into gray matter, white matter, and cerebrospinal fluid, the gray matter images were used to determine the normalization parameters for the standard MNI gray matter template. At this point, the spatial parameters were applied to the slice-timed and realigned functional volumes that were resampled to 2.0 x 2.0 x 2.0 mm voxels and smoothed with a 6-mm FWHM kernel. Each voxel’s time series was corrected for noise using the SPM standard 128-s high-pass filter combined with AR auto correlation correction modeling. In addition, we employed voxel wise multidimensional regression analysis in a standardized manner to remove artifacts resulting from motion and changes in ventricle and white matter signals (Verhagen et al. 2006), by adding 18 movement regressors; 6 parameters obtained from rigid-body head motion correction (SPM 8 statistical package), and their squares and cubes.

**Venous blood sample**

Venous blood samples were only collected from HoM, (for another study, and have normal levels). Venous blood samples were collected between 8 and 10 a.m. in the morning.

Plasma testosterone levels (nmol/L), (radioimmunoassay, Testosterone RIA DSL-4000, Diagnostic Systems Laboratory Inc., TX), and the sex hormone binding globulin (SHBG) were analyzed in the Chemical Diagnostics Laboratory at Karolinska University Hospital. The levels of bioavailable testosterone (nmol/L) were calculated using an equation developed by Sodergard et al. (Sodergard et al., 1982). Also the oestradiol, Follicle stimulating hormone (FSH) and luteinizing hormone (LH) levels were analyzed in the Chemical Diagnostics Laboratory at Karolinska University Hospital.

**Social Responsiveness Scale (SRS)**

SRS is a questionnaire commonly used for evaluating autistic traits (Constantino and Gruber, 2005). It was administered to our three populations in regard to their potential participation in another project. The scores are presented here as they are relevant to a discussion of the factors that could potentially bias the present results. The SRS required that someone close to the participant complete it, such as a parent, partner or close friend. The 65-item SRS questionnaire is scored on a scale from 0 (never true) to 3 (almost always true). Total scores can range from 0 to 195. Lower scores on the questionnaire signify less autistic behavior over the 6 months prior to the testing, whereas higher scores signify more autistic behavior over this period. Normal range for SRS T Points is 59 or less, and none of the subjects scored above this value.

**S1 Fig. Group differences in cortico-cortical covariations of Cth from the precuneus ROI.**

The scale is is logarithmic and shows log10(P); Warm colors indicate positive contrast and thus greater covariation in HoM than controls (HeM and HeW). Clusters calculated at p<.05 after Monte Carlo correction are superimposed on a standard MRI brain

**S2 Fig. Correlation between Kinsey scores and cortical thickness**

Sagittal view of the standard brain MRI (atlas retrieved from the FreeSurfer program’s pipeline), showing regions in which Kinsey scores were significantly correlated with Cth. Scale is logarithmic and shows –log10(P), with warm colors positive correlations (thicker cortex, higher Kinsey score), cool colors indicating negative correlation.
