# Supplementary material for: Multimodal MRI suggests that male homosexuality may be linked to cerebral midline structures
Source: PLoS One. 2018 Oct 2;13(10):e0203189. doi: 10.1371/journal.pone.0203189 (PMC6168246; doi:10.1371/journal.pone.0203189)
Supplement: S1 Table — (DOCX) [file pone.0203189.s004.docx]

**S1 Table. Scores in a test of social functions**

|  | | HeM (N= 35) | | HeW (N= 38) | | HoM (N= 30) | | F*(df*)-value *P*-value |
| --- | --- | --- | --- | --- | --- | --- | --- | --- |
|  |  | Mean | Sd | mean | Sd | Mean | Sd |  |
| SRS t-point |  | 42.8 | 6.1 | 45.9 | 7.5 | 47.4 | 7.3 | 2.8(*2,100)*  .68 |

F-values from group comparisons (one way ANOVA). Possible differences between the separate groups were calculated with Scheffe’s post hoc test (p<.05).

SRS t-points are from the social responsiveness scale, Constantino & Gruber, Social responsiveness scale. Los Angeles: Western Psychological Services.
