# Supplementary material for: Multimodal MRI suggests that male homosexuality may be linked to cerebral midline structures
Source: PLoS One. 2018 Oct 2;13(10):e0203189. doi: 10.1371/journal.pone.0203189 (PMC6168246; doi:10.1371/journal.pone.0203189)
Supplement: S2 Table — (DOCX) [file pone.0203189.s005.docx]

**S2 Table. Hormone levels in HoM (n=30)**

|  |  |
| --- | --- |
| Testosterone (nmol/L)  active testosterone  Oestradiol (nmol/L)  FSH (nmol/L)  LH (nmol/L) | 9.5±0.5  5.4±0.5  <150  6.6±2.1  5.3±1.3 |
